# Flanking regions, amyloid cores, and polymorphism: the potential interplay underlying structural diversity

**DOI:** 10.1016/j.jbc.2023.105122

**Published:** 2023-08-01

**Authors:** Anukool A. Bhopatkar, Rakez Kayed

**Affiliations:** 1 Mitchell Center for Neurodegenerative Diseases, University of Texas Medical Branch, Galveston, Texas, USA; 2 Departments of Neurology, Neuroscience and Cell Biology, University of Texas Medical Branch, Galveston, Texas, USA

**Keywords:** Neurodegeneration, amyloid polymorphism, intrinsically disordered proteins, protein aggregation, protein folding, amyloid-beta, alpha-synuclein, tau protein, TDP-43

## Abstract

The β-sheet–rich amyloid core is the defining feature of protein aggregates associated with neurodegenerative disorders. Recent investigations have revealed that there exist multiple examples of the same protein, with the same sequence, forming a variety of amyloid cores with distinct structural characteristics. These structural variants, termed as polymorphs, are hypothesized to influence the pathological profile and the progression of different neurodegenerative diseases, giving rise to unique phenotypic differences. Thus, identifying the origin and properties of these structural variants remain a focus of studies, as a preliminary step in the development of therapeutic strategies. Here, we review the potential role of the flanking regions of amyloid cores in inducing polymorphism. These regions, adjacent to the amyloid cores, show a preponderance for being structurally disordered, imbuing them with functional promiscuity. The dynamic nature of the flanking regions can then manifest in the form of conformational polymorphism of the aggregates. We take a closer look at the sequences flanking the amyloid cores, followed by a review of the polymorphic aggregates of the well-characterized proteins amyloid-β, α-synuclein, Tau, and TDP-43. We also consider different factors that can potentially influence aggregate structure and how these regions can be viewed as novel targets for therapeutic strategies by utilizing their unique structural properties.

The amyloid form of proteins has been implicated in multiple pathological conditions, predominantly being associated with neurodegenerative disorders where proteinaceous deposits are ubiquitously observed in the brain of afflicted individuals (1, 2). Clinical and experimental evidence has established a firm correlation between the presence of protein aggregates and the universal toxic sequalae seen in neurodegenerative disorders, which lead to widespread neuronal dysfunction and gradual atrophy (1, 2, 3). Contrastingly, the amyloid state is also utilized in certain instances by organisms for physiological functions such as the Curli amyloid, used by bacteria for biofilm formation, and Sup35, that functions in yeast translational regulation (4, 5). A provocative idea that has recently emerged in the field is that amyloid structures of proteins might have crucial role in neuronal functioning by serving as engrams which purportedly can encode memories, one example being the Orb2 amyloid in *drosophila* (6, 7). Following the initial identification and characterization of Amyloid-beta protein (Aβ) associated with Alzheimer disease (AD) in early 1980s (8, 9), other proteins associated with distinct neuropathologies were also identified. These include the microtubule-associated Tau protein, also implicated in AD and other degenerative pathologies termed as tauopathies, and the Prion protein (PrP) associated with Scrapie and Creutzfeldt-Jakob Syndrome which were also identified during the same time period. Identification of other proteins soon followed; Fused in Sarcoma (FUS) and TAR DNA-binding protein-43 (TDP-43), both linked to amyotrophic lateral sclerosis (ALS), α-Synuclein (αSyn) in Parkinson’s disease (PD), multiple systemic atrophy (MSA) and Lewy body dementia (LBD), and huntingtin protein in Huntington disease, among many others. Despite obvious differences in the amino acid sequences, studies over the past 2 decades have shown that these proteins show a remarkable convergence onto a generic structure, termed as the amyloid motif, irrespective of their sequence, suggesting common underlying principles guiding their formation (2, 10, 11, 12, 13). The overall amyloid fold is defined by an orderly, linear arrangement of individual units of a protein whose β-strands are oriented perpendicular to the chain axis, adopting the hallmark cross-β sheet structure (2, 10, 11, 12, 13, 14).

The inceptive research on amyloids was concerned with the high-molecular weight fibrils which were evident in histopathological examinations and hence were considered the primary pathogenic species (9, 15, 16, 17). However, clinical and experimental observations have led to a re-evaluation of this paradigm as cognitive and behavioral deficiencies appeared in patients without significant fibrillar burden (18, 19, 20, 21). The focus was soon shifted to the intermediate species in the aggregation pathways, which were then posited to play pathologically relevant roles (20, 21, 22, 23). Among them, a large body of research has shown that the oligomeric form of proteins can induce significant toxicity in cellular and animal models (21, 23, 24, 25, 26, 27). A prominent reason for the potent toxic effects of oligomers is their soluble, diffusible nature, which allows them to spread from cell-to-cell and eventually to different anatomic regions in the brain, propagating their toxic fold, thus exhibiting prion-like behavior (21, 28, 29, 30, 31, 32). Furthermore, oligomers can disturb the integrity of cellular membranes and initiate myriad cytotoxic sequalae (21, 33, 34). Being cognizant of this new knowledge about oligomers, protofibrils, and fibrils, investigators have since focused on characterizing the entirety of the heterogenous species within the amyloid pathway in hope of developing therapeutic strategies to combat these fatal disorders. These studies have been spurred on by rapid developments in the tools and techniques available, namely solid-state NMR and cryo-EM (35, 36, 37, 38, 39, 40, 41, 42), to interrogate the amyloid structures at the molecular and atomic scales.

High-resolution studies of amyloids have enabled us to interrogate their subtleties and identify the structural variants of their aggregates (43). These variants, formed from a single protein with the same primary sequence, are termed as polymorphs. Structural polymorphs of a protein adopt the hallmark cross-β sheet fold but show differences in their overall morphology, symmetry about the axis, and side-chain arrangements (43, 44). Such diversity is evident in the fibrillar, proto-fibrillar, as well as oligomeric forms of the protein (43). The origin of such conformational variety in amyloid structures is one of the most relevant topics of study in the field today, as it is hypothesized to induce unique pathological cascades and give rise to phenotypic variations in neurodegenerative disease. We know that the final conformation assumed by the amyloid form is a direct consequence of the monomeric properties and unique conditions encountered during the initial misfolding events. Among the many factors that potentially influence monomer dynamics, the cellular stimuli and environmental variations surrounding the monomer have been prominently looked at.

When parsing through current literature, it is evident that regions adjacent to the amyloid core play a role in physiological functions of native proteins and, crucially, in their pathological transition to the amyloid form. As reviewed by Ulamec *et al*. (45) and Gallardo *et al.* (46), the structural and functional properties of amyloid-flanking regions widely affect the process of protein aggregation. Our goal here is to cumulate available evidence to highlight the potential role played by amyloid-flanking regions in inducing structural polymorphism. We first look at the process of amyloid formation and review the structural and functional properties of disordered regions of the well-characterized amyloidogenic proteins: Aβ, αSyn, and Tau, along with TDP-43, a protein whose amyloid fibril–forming potential is still debated. This will be followed by a review of their polymorphic aggregates. Finally, we contemplate the potential of these flanking regions as therapeutic targets. Although there are countless amyloid-forming proteins that have been identified, we chose to limit the scope of this review only to those that are involved in neurodegenerative diseases (47, 48).

## The process of amyloid formation

The amyloid state represents one of the most unique structural motifs in proteins. Although we associate the proteins that form this structure with pathology, it putatively represents the most energetically stable conformation for any protein (44, 49, 50). This hypothesis is echoed in the conformational commonality we observe in most amyloid proteins. Because of the progress in computational modeling and bioinformatic algorithms, we can leverage our theoretical understanding to predict the propensity of a given protein sequence to form amyloids with a high success (51, 52, 53, 54, 55, 56, 57, 58). A common theme underlying amyloidogenic proteins is a lack of secondary structural features within their sequences. Prominent examples are Aβ (59), Tau (60), αSyn (61), and PrP (62), whose monomeric form is predominantly devoid of defined structural features. Proteins which lack a well-defined secondary structure throughout the entirety of their sequence are termed as intrinsically disordered proteins (IDPs), while smaller segments are called intrinsically disordered regions (IDRs) (63). Such peptides demonstrate significant flexibility which allows them to sample an ensemble of different structures (63, 64, 65). Reports have shown that this conformational freedom is also responsible in making these proteins prone to aggregation (65).

Our current understanding of amyloid formation is based on *in vitro* experiments with simplified systems that suggest the process can be broadly understood using the nucleation-dependent polymerization model (66, 67). The model posits that the process is initiated by a misfolding event of the disorder-rich protein monomer, which then serves as a nucleus, or a template, for misfolding of other monomers, akin to crystallization (2, 68, 69, 70). The formation of such a nucleus is termed as nucleation, and it happens in a period called the lag time, along with multiple other events that eventually determine the fate of the aggregation pathway. Details about the multitude of microscopic processes that define this period are given in excellent reviews by Michaels *et al*. (71) and Arosio *et al*. (72). Once a stable, misfolded nucleus has formed, there is rapid addition of monomers and propagation of the template conformation (73). Recent reports, however, suggest that this model requires certain modifications to capture the extensive variety of aggregation dynamics and pathways utilized by different proteins (73, 74, 75, 76). Modification to this model assumes a multistep process requiring a conformational conversion to form an intermediate species. This model, called the nucleated-conformational-conversion (NCC) model, better explains the observed formation of stable intermediate oligomers without a dependence on monomer concentration. The validity of this model was demonstrated in the prion-like yeast protein, Sup35, where kinetics of protein aggregation necessitates the formation of an oligomeric intermediate in the assembly process (77). Such a mechanism was also found to be active in fibrillation of Aβ42, where low-molecular weight oligomers serve as seeds for aggregation (78). The final conformation adopted by the amyloid form is observed to emerge early in the aggregation pathway and is then propagated (79, 80, 81, 82). One can then hypothesize that the discrete events occurring early within nucleation imprint themselves on the final amyloid structure. The unstructured nature of the monomer affords it a conformational freedom to sample multiple, thermodynamically equivalent states until a metastable form is adopted (82). Aggregation to oligomeric and fibrillar stages proceeds when a sufficient number of monomers assume a metastable state capable of propagation (82, 83, 84, 85). The NCC model, although it explains the vast majority of aggregation kinetics for different proteins, is not the only accepted model that describes experimental results and, considering the variety of amyloidogenic proteins and the environmental conditions they encounter, it is likely that one simple model will not suffice (86, 87). Additionally, we would like to highlight reviews by Kelly *et al*. (88), Chatani *et al*. (74), Lasagna-Reeves *et al*. (89), and Serio *et al*. (77) for excellent description and depiction of amyloid aggregation models (including NCC).

In studies on Aβ40 aggregation, Sciarretta et al. synthesized a congener Aβ40 peptide that contains a lactam bridge between the side chains of Asp23 and Lys28 (90). This model peptide showed significantly higher aggregation kinetics as it bypasses unproductive folding states and rapidly forms a stable nucleus capable of propagation (84, 90). The growth of the oligomeric form is dictated by the lock-and-dock mechanism where addition of new monomers requires conformational corruption (82, 85). Such a mechanism is postulated to underlie most amyloid oligomer elongation (82). The addition of monomers and growth of aggregates often involves expulsion of water, mostly as a consequence of the stabilizing interactions and assumed conformation (82). As oligomers grow, the β-sheet content of the structure grows linearly with conformational rearrangement from antiparallel to parallel β-sheet arrangement (91). Unlike the fibrils, the oligomeric state of amyloids resists strict definitions and is context-dependent, varying in size and conformational features (92, 93). Depending on the kinetic and thermodynamic stability of the intermediate oligomers formed, the folding route (or in this case, misfolding) accessed by the monomer is termed as on-pathway or off-pathway (85, 94, 95). The on-pathway is characterized mostly by ephemeral intermediate species which rapidly convert to fibrillar structures, while the off-pathway is defined by the presence of relatively stable, kinetically-trapped intermediate forms which persist as such or dissociate into monomers (85, 95). Although this distinction is helpful in coarsely demarking the pathways utilized by the monomer en route to amyloid formation, one should keep in mind that these definitions are not strict and exhibit a degree of ambiguity and that there is interconversion of species from one pathway to another (85, 94). The oligomeric state is the precursor to protofibrils, which represent structurally mature amyloid species on-pathway to fibrils. The demarcation of protofibrils and fibrils is usually dependent upon their size and the equilibrium maintained with other products of the aggregation pathway (96, 97).

**Figure 1:**
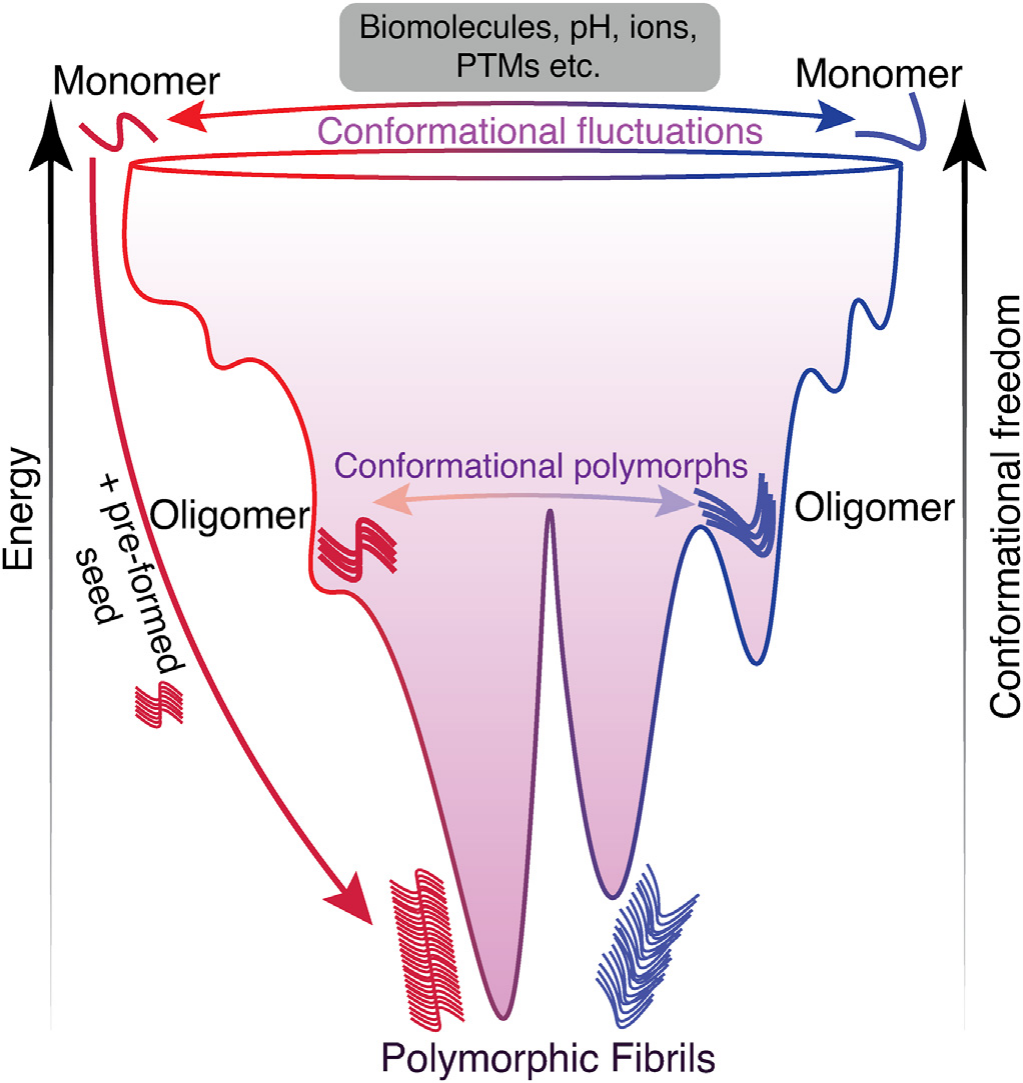
**Generation of polymorphic amyloid aggregates**. A schematic depicting the protein folding funnel for aggregation of amyloid proteins. Polymorphism of amyloid oligomers and fibrils is a consequence of the unique misfolding pathway the dynamic, high-energy protein monomers undertake. The monomeric form of the protein can undergo conformational fluctuations in the presence of certain cellular conditions and factors, along with any PTMs on the monomer, leading to the adoption of distinct conformational states. Most of the modulatory factors (PTMs, interactions with other biomolecules) discussed here are relevant in physiological conditions (*in vivo*) and are usually absent from *in vitro* experiments probing protein aggregation. The propagation of a distinct state can then lead to polymorphic oligomers and fibrils. Additionally, this intervening aggregation pathway can be bypassed *via* the addition of a preformed fibrillar seed. PTM, posttranslational modification.

For our purposes here, we use a simplistic description of the process without delving into its complexities, such as secondary nucleation, fragmentation, or role of biomolecular phase transitions in amyloid formation (Fig. 1). Using a protein-folding funnel, one can view the monomeric ensembles undergoing conformational interconversions due to various factors (discussed in detail below), eventually self-nucleating their assembly to oligomers and fibrils, the final conformations of which depend on the exact route taken (Fig. 1). For a detailed, quantitative explanation of amyloid formation, readers are directed to excellent reviews by Dobson and Chiti (2), Morris *et al*. (73). The entire spectrum of mechanisms governing this process remains under investigation, but sophisticated modeling algorithms are constantly updating our knowledge (67, 74, 82).

## Structural disorder and flanking regions of amyloid cores

The paradox of amyloid proteins is that their ordered, structured core is often flanked by disordered, flexible regions (45, 98, 99). Structural disorder within amyloid proteins is recognized as one of the driving factors that accommodates their structural transitions to the β-sheet–rich form (98, 99). The significant entropic contributions of these regions compensate for the extremely ordered and rigid amyloid structures that form. Beyond this pathological association, disorder in proteins is a factor that imbues them with functional promiscuity (63, 64, 100, 101). Indeed, IDPs are recognized to play central roles in multiple signaling and interaction pathways (64, 100, 101, 102, 103, 104). The multifunctional nature of IDPs and IDRs is a direct consequence of their structural dynamism which allows interaction with multiple partners. Such disordered proteins and regions are characterized by enrichment in polar and charged amino acids, categorized as disorder-promoting, while being devoid of hydrophobic, order-promoting amino acids (105, 106, 107). And thus, the sequences of IDRs and IDPs show a compositional bias and have less diversity, commonly containing repeat modules (101, 105, 107, 108).

Despite the lower amino acid diversity, disordered proteins display a rich and complex structural variety. These proteins have a relatively flat energy landscape and can access an ensemble of energetically equivalent, interconvertible conformations (109, 110). Structural fluctuations on a spatiotemporal scale are common and dictated by a variety of factors such as sequence properties (111, 112) and the cellular environment (110, 112, 113). IDPs and IDRs are also known to attain structural order upon interaction and binding to specific partners (114, 115, 116). On the other hand, some IDPs are shown to retain structural disorder despite showing high-affinity binding to their ligands, suggesting a defined binding site is not mandatory for interaction (117). The binding-induced folding of IDPs represents an active area of research, as it provides an explanation for the multifunctional nature of these molecules. With help of modern computational tools, we can tease out the general characteristics of disordered regions involved in binding and folding processes. Based on such investigations, we currently recognize three distinct disordered regions that serve as interaction modules: molecular recognition features (MoRFs), short linear motifs (SLiMs), and low-complexity regions (118, 119, 120, 121). Each module displays a characteristic feature, such as length and selectivity for interaction partners, and serves as an indicator for functional capabilities of the protein (118, 119, 120). Recently, a study investigated the structural disorder in the proteins involved in the Aβ cascade, finding that the amyloid precursor protein and ApoE, along with others, show a strong propensity for structural disorder in the form of MoRFs (122). Such findings highlight the prevalence of disordered interaction modules in pathophysiology and the need to better understand their behavior. To explain the disorder-order transition for these regions and proteins, the induced fitting model and conformational selection models, or a combination of the two, are put forth (116, 123). The induced fitting model suggests that an unstructured protein chain can attain a fold that is energetically accessible and allows a productive interaction, while the conformational selection model postulates that only those unfolded protein chains that are in a conducive structural state prior to interaction are ‘selected’ for binding. However, the staggering diversity of disordered proteins and their ensembles means that it is likely there are multiple mechanisms at play. Such a variety is evident in protein–nucleic acid interactions where disordered regions such as SLiMs and low-complexity regions represent the predominant modules used by proteins to bind nucleotides (118, 119, 124, 125). In this respect, the review by Lee *et al*. (126) represents an excellent compendium to understand the diversity in disordered sequences in proteins, their classification, and their characteristics.

Along with the multitude of interaction partners, cellular conditions also heavily modulate structural aspects of disordered proteins. While reviewing available data for the factors modulating protein conformations (pH changes, ionic concentrations, posttranslational modifications (PTMs)), we need to bear in mind that most of the evidence comes from experiments performed *in vitro*, but we can extend these principles and observations to the cellular environment. Early investigations of αSyn, an IDP associated with multiple neurodegenerative disorders, identified that pH is an important modulatory factor of its conformational state (127). The authors identified that an acidic environment (low pH) generated a misfolded intermediate of αSyn that had a higher aggregation propensity which formed morphologically distinct fibrils (127). Another example of this is seen in human insulin, where pH changes induce polymorphism in the amyloid structures with distinct spectroscopic signatures (128). A recent study on prothymosin, in which the authors identified that an acidic environment leads to compaction of the protein chain and enhances its ability to form β-sheets (129), further highlights the effect of environmental pH on protein conformation. Multiple other reports have established the prominent role of pH in the aggregation and conformational state of disordered proteins; depending on the exact sequence, a protein chain can undergo collapse or expansion to attain energetic equilibrium (130). In a similar manner, the electrostatic interactions with cellular ions also affect the conformational state of IDPs and IDRs (131). This would be expected based on the preponderance for charged amino acids to engage in coulombic interactions with each other and with the surrounding environment. The effect of ions on disordered proteins and their conformational state is highly context-dependent; the number of charged amino acids that can engage in interactions, their patterning, and the exact concentration and ionic composition of the solvent, all play a role (131, 132, 133, 134). Investigating the effect of salt concentration on αSyn, Roeters et al. (135) found a striking difference: low salt buffers induce the formation of extended, parallel β-sheets, while high salt conditions induce a compact, antiparallel β-sheet structure in the fibrils.

Additionally, unstructured regions in proteins also serve as regulatory hubs *via* PTMs. The high solvent exposure of these regions allows easier access to any modifying enzymes, adding a further level of complexity to the structural-functional landscape of IDRs and IDPs. Since the properties of IDPs and IDRs are so intricately linked to hydrophilic residues of which they are composed, they can be drastically altered by any modification. Among the myriad PTMs, phosphorylation is observed to be the most common one (136), especially for IDPs and IDRs (137). A comprehensive study by Iakoucheva *et al*. (138) revealed that most phosphorylation sites and adjacent regions have properties associated with disordered segments, suggesting phosphorylation might be a very ubiquitous and powerful tool to modify IDPs/IDRs. For disordered regions, addition of a negative charge in the form of a phosphate group can enhance or attenuate interactions and alter the entire structural conformation (137, 139). The results of the meta-analysis performed by Darling *et al*. reveals a strong association between intrinsic disorder in proteins and other PTMs like amidation, ubiquitination, sulfation, prenylation, myristoylation, and glycation (137). These observations underline the importance of IDPs/IDRs as multifunctional hubs that can be controlled *via* multiple cues depending on cellular needs.

**Figure 2:**
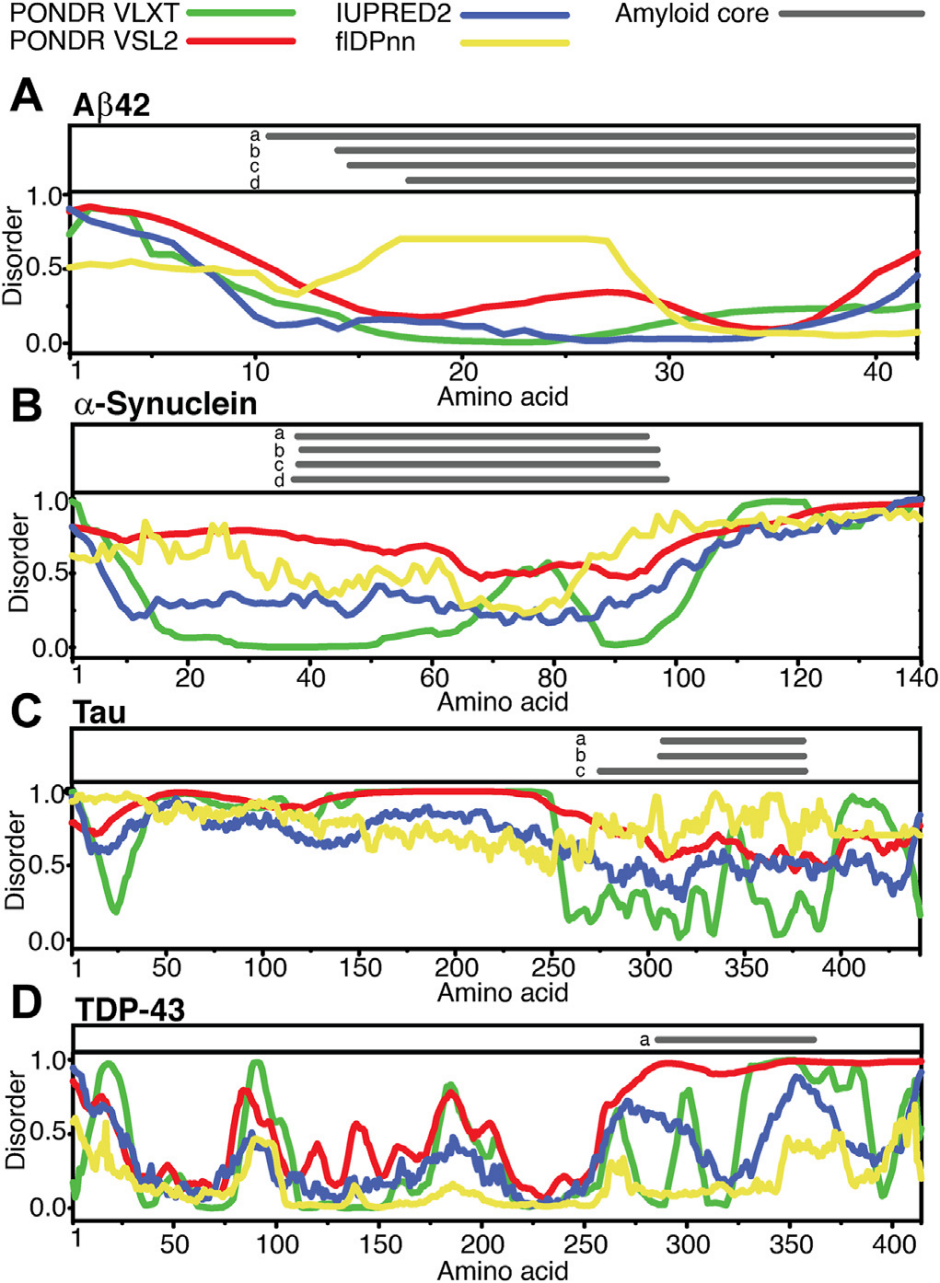
**Disorder propensity in amyloid proteins**. The disorder propensity of well-characterized amyloid proteins Aβ, α-Syn, Tau, and TDP-43 is predicted using four computational algorithms: PONDR VLXT(151) (*green*), PONDR VSL2 (151) (*red*), IUPRED2A (315) (*blue*), and flDPnn (152) (*yellow*). The disorder in protein sequence is assigned a value from 0 to 1, with a higher value indicating a high confidence prediction of disorder. Above the disorder plots are experimentally verified amyloid core regions for each protein indicated with *gray* horizontal lines: *A*) Aβ: a (154), b (153), c (156), d (155); *B*) α-Syn: a (172), b (169), c (171), d (170); *C*) Tau-2N4R: a (186), b (185), c (264); and *D*) TDP-43: a (201). Aβ, amyloid beta; TDP, TAR DNA-binding protein.

A hallmark feature of IDPs and IDRs is their poorly defined presence or complete absence in electron density maps in X-ray crystallography experiments. Consequently, their early identification in proteins was inferred by subtraction from solved structures of ordered regions (140, 141). Similar bias underlies the predictions of IDPs and IDRs by computational algorithms which use the protein data bank (PDB) library as their primary training dataset (X-ray structures make up 89% of the PDB database) (141, 142, 143). The use of complementary datasets from techniques such as CD, NMR, and cryo-EM has the potential to alleviate this bias (141, 142). Unlike X-ray crystallography, these techniques allow one to directly discern the unique signatures of IDPs and IDRs (144). Rapid advances in artificial intelligence and machine learning promise to provide highly accurate structure prediction for proteins, including disordered ensembles (145, 146, 147). Here, we evaluated the structural disorder within the well-characterized amyloidogenic proteins Aβ42, αSyn, Tau-2N4R, and TDP-43 using the computational tools IUPRED2A (148, 149), PONDR-VLXT, PONDR-VSL2 (108, 150, 151), and flDPnn (152) (Fig. 2).

We chose these algorithms since they utilize discrete strategies to generate sequence-disorder predictions; IUPRED utilizes the composition and arrangement of amino acids in a protein to estimate a pairwise energy. This energy represents the basic parameter which is utilized by the algorithm in predicting whether a given protein has a stably folded structure with an energy minimum (149). IDPs and IDRs show characteristic pairwise energy values which are used to distinguish them from globular sequences (149). The PONDR platform utilizes neural network predictors, which were trained on various datasets of ordered and disordered sequences. These neural network predictors scan varying length of sequences of an input protein with preassigned attributes for individual amino acids. The individual predictors (VLXT, VSL2) differ in terms of the datasets utilized in their training and their scan window in each prediction. Finally, the flDPnn server predicts disorder in a protein sequence *via* a three-step model in which the first step relies on multiple other disorder-predicting algorithms to generate a disorder profile of the input sequence (152). The second and third steps rely on a deep machine-learning algorithm to provide final quantification of sequence disorder (152). This server can also provide accurate predictions of function based on sequence properties and disorder scores (152). As is evident from the underlying processes of these algorithms, one must practice caution when interpreting disorder predictions as most training datasets used will include the proteins that serve as the input in our case. Accordingly, the known amyloid cores will expectedly have low disorder prediction, even though these sequences might be disordered prior to aggregation. Our aim remains to investigate the presence or absence of disorder within the regions flanking the amyloid cores. Consequently, we also parsed the PDB library for experimentally verified amyloid core sequences to look at the juxtaposition of structural disorder in proteins and their amyloid-forming regions.

### Aβ42: flanking regions: aa 1 to ∼15 (153, 154, 155, 156)

All computational platforms used here predict lower disorder scores for regions encompassing the amyloid core, as expected, while a high disorder score is assigned to flanking regions (Fig. 2). In case of Aβ42, low disorder scores are assigned to the amyloid core–forming region extending from the middle of the protein to the C-terminal end (∼aa 15–42) by three predictors: VLXT, VSL2, and IUPRED2A, while for reasons we are unsure of, flDPnn assigns a higher disorder score to central region of the protein. However, all four platforms expect the N-terminal region to contain higher disorder (Fig. 2*A*). Empirical findings on the truncated forms of Aβ42 reveal that the disordered flanking region also has reduced aggregation propensity, while the more ordered amyloid core–forming region is aggregation-prone. Liu *et al*. (157) identified Aβ_11-42_, an N-terminal truncated fragment of Aβ, in the brain extracts of AD patients; this fragment shows similar aggregation propensity as the full-length protein and forms insoluble deposits. In contrast, Mazzitelli et al. (158) observed that, while the C-terminal truncated fragment of Aβ, Aβ_1-24_ (devoid of C-terminal), is capable of cross-seeding the aggregation of Aβ42, it has attenuated ability to form aggregates by itself. The precise physiological roles of Aβ remain unknown, although a few putative functions suggested are antimicrobial activity, tumor suppression, synaptic modulation, among others (159). In terms of PTMs of Aβ42, most of those are observed to happen on the disordered, N-terminal region of the protein (160). Aβ undergoes phosphorylation at S8 along with nitration and glycosylation at Y10 (160). These modifications are postulated to enhance the amyloid formation of Aβ (161). Additionally, longer forms of Aβ peptide (Aβ43–49) are also formed due to the promiscuity in secretase proteolysis (162, 163, 164) and have been identified in a pathological state (163, 165, 166). Characterization of the longer forms of Aβ (Aβ43-Aβ49) reveals a similar aggregation propensity for these proteins, as well as cross-seeding ability (167, 168). Although detailed structural insights on the amyloid core formed from the longer variants are absent, we can speculate that the extended tail might potentially represent another flanking region for Aβ.

### αSyn: flanking regions: aa 1 to ∼35, ∼100 to 140 (169, 170, 171, 172)

For αSyn, a higher disorder score is assigned by all four predictors to the C-terminal end, while lower disorder values are predicted for the rest of the protein, including the amyloid core–forming nonamyloid component domain and the N-terminal region (Fig. 2*B*). The amyloid core of αSyn is observed to extend from amino acid ∼35 to 100 (169, 170, 171, 172). This N-terminal domain of αSyn is known to undergo a transition to an α-helix to enable interaction with phospholipids in the cell membrane (173), while the C-terminal domain serves as the interaction module for other protein in its multiple physiological roles (174). Studies on αSyn fragments show that only the full-length protein shows prominent aggregation capabilities, while the truncated forms did not form detectable, thioflavin-T positive aggregates (175). In fact, results have ascribed a protective effect to the C-terminal domain *via* attenuation of αSyn aggregation potentially by enhancing its interactions with other biomolecules (176, 177, 178). PTMs also modify αSyn, and many are associated with pathology (179, 180). Phosphorylation of αSyn is localized to the disordered C terminus, specifically on the residues Y125, S129, Y133, Y135 (179). Among these, S129 has emerged a potential biomarker for synucleopathies, although it remains unclear precisely what consequences this modification has on the aggregation of the protein (181). Additionally, αSyn also shows prominent ubiquitination of lysine residues in the N-terminal region (179, 182).

### Tau 2N4R: flanking regions: aa 1 to ∼300, ∼380 to 441 (183, 184, 185, 186)

We see replication of above trends with Tau 2N4R; all platforms predict a high degree of disorder in most of the protein (the projection domain which includes the N-terminus and central region) apart from the C-terminal region (Fig. 2*C*). This region corresponds to the microtubule-binding domain (MTBR) containing the 4R pseudo-repeats, which reports show is intrinsically disordered (187, 188). As stated before, this observation brings out the perplexity in disorder prediction algorithms since they anticipate regions of order based on prior experimental data, which in this case assigns order to the MTBR since it eventually forms fibrils. As seen in Figure 2*C*, the amyloid core of Tau also lies in the MTBR, leading to a loss of function upon aggregation. To this point, a recent report by Elbaum-Garfinkle and Rhoades looked at the effect of the anionic molecule heparin on the conformation of Tau monomer using a FRET assay (189). The results indicated that the C terminus of Tau undergoes compaction, while the disordered N terminus, which serves as the interaction hub for Tau, maintains an extended form. The authors identify this conformational state of Tau as aggregation prone (189). Studies on truncated forms of Tau reveal the MTBR is self-sufficient in aggregation (190, 191, 192). Along with its association with the cytoskeletal elements, Tau also binds and interacts with nucleic acids, chaperones, membrane proteins, kinases, and phosphatases (193). The N-terminal and proline-rich regions within the projection domain of Tau serve as interface for these interactions (193). The most prominent PTM of Tau is phosphorylation, which is abundantly observed in AD pathology, predominantly being modified in the proline-rich region and C-terminal end of the protein (186, 194). In AD, phosphorylation is prominently observed at Y18, S199, S202, T205, T231, S262, S396, and S422, with the pattern showing a spatio-temporal dependence (195). Three phosphorylation sites that have shown potential to serve as biomarkers for AD progression are T181, T217, and T231 (194, 196, 197, 198). The hyperphosphorylated form of Tau is unable to bind to microtubules and leads to loss of function and gain of toxic function in the form of aggregates. Other PTMs observed in Tau are ubiquitination, acetylation, glycosylation, nitration, and SUMOylation, specifically depending on the pathology and isoform (199, 200).

### TDP-43: flanking regions: aa 1 to 280, 361 to 414 (201)

Finally, TDP-43 is unique among the proteins discussed here for two reasons: firstly, there is ambiguity with regards to whether TDP-43 can form genuine amyloid fibrils, as majority of the studies report an amorphous nature (201) and secondly, it is predicted to show longer stretches of disorder within its amyloid-like (we chose to describe the amyloid core as amyloid-like) core-forming region, whereas the rest of the protein has a lower disorder score (Fig. 2*D*). The amyloid-like core region of TDP-43 corresponds to the well-characterized disordered, low-complexity domain (LCD) of the protein (202, 203). Remarkably, this entire stretch of the protein (amino acid 267–414) is capable of transitioning to an amyloid structure, as described by Li *et al*. (204). The C-terminal fragments of TDP-43 have been ubiquitously observed in ALS-frontotemporal dementia (FTD) patients and represent a pathological marker (48, 205). Among these, TDP-43 C35 and C25 (C-terminal fragments of 35 kDa and 25 kDa, respectively) are prominent (48). The sequences of TDP-43 C35 and C25 consist of two- and one-RNA recognition motifs, respectively (205). The N-terminal domain of TDP-43 alone is also sufficient for oligomer formation (206), without necessarily adopting an amyloid state (207). Based on these observations, it is suggested that the N-terminal domain assists and enhances the aggregation of the C-terminal region *via* its oligomerization ability (207, 208). Functionally, TDP-43 is an RNA-binding protein and is known to play multiple physiological roles, most important being its splicing and translational regulation (209). The protein also plays a role in cellular stress response in the form of stress granules, which represent protective assemblies of RNA-binding proteins and nascent mRNA (210). The LCD of TDP-43 is enriched in phosphorylation sites (211) with those at S409 and S410 being ubiquitous in TDP-43 pathologies (212). Despite the hyperphosphorylated state being observed in pathology, there is still ambiguity about the role of this PTM in eliciting toxicity. Additionally, the RNA recognition motif (RRM) of TDP-43 can be acetylated in response to cellular stress, leading to abrogation of RNA binding and induction of protein aggregation (213).

The presence of disordered segments has also been verified in other proteins such as FUS (214) and TIA-1 (215), associated with ALS; PrP, associated with Creutzfeldt-Jakob syndrome (216, 217); Musashi-1,2, associated with AD (218, 219); and Huntingtin, associated with Huntington’s disease (220). Pathologically, these proteins also affect their toxicity through the formation of amyloid aggregates, highlighting a trend between protein disorder and amyloid formation.

## Conformational dynamics and polymorphism of amyloid structures

The fibrillar form of amyloids represents the archetype of protein aggregates due to their size and hallmark structure. Despite the adoption of a common overall fold (cross β-sheet), the heterogeneity in amyloid structures is now well established (42). Such structural variation purportedly underlies distinct phenotypes observed in neurodegenerative disorders (43, 44). Similarly, or perhaps more, structural variations are possible for the oligomeric form, allowing a staggering number of possible polymorphs. Amyloid oligomers are known to display diversity in their overall tertiary and quaternary morphology, such as being spherical, micellar, disc-shaped, annular, and others (59, 221, 222, 223). They also display differences in their secondary structure, from the adoption of antiparallel β-sheet (135, 224), parallel β-sheet (225), or α-helical (226) structure, to an unusual α-sheet structure (227), whose existence was first postulated by Pauling and Corey in 1951 (228). The oligomeric form of the protein is not always rigid, and many examples have revealed noteworthy conformational flexibility within these species (91, 93, 229, 230, 231, 232). The relationship between such dynamic behavior of protein aggregates and their final form is unclear. It is also unknown how the structural disorder within the amyloid core and adjacent regions accommodates any conformational transitions. One reason for the dearth of information is the transient, metastable nature of these intermediate species. Such transitions are observed to occur on a temporal scale of micro- to milli-seconds (93). This makes computational modeling an invaluable tool in such studies, alongside NMR (93). In a recent review by Sun *et al*., the authors outline observations from various results on dynamics of amyloid oligomers along with the experimental techniques used to interrogate them (93). Having looked at the structural disorder in the sequences of four amyloid proteins, we will review conformational dynamics and polymorphism within their aggregates. For further reading, we bring to attention informative reviews on amyloid oligomers by Sengupta *et al*. (28) and Gerson *et al*. (233), which focus on providing an overview of Aβ and Tau oligomers.

### Aβ: oligomer dynamics

Most of our knowledge regarding the aggregation kinetics and dynamics of amyloid formation is obtained from Aβ, which formed the focus of intense studies at the advent of the field and still represents an attractive model because of its well-characterized aggregation kinetics. The insights and understanding gleaned from this pioneering research have been extended to other amyloid proteins. Early experimental investigation on Aβ aggregation dynamics using NMR revealed that monomeric states that had more structural disorder, or lower energy barriers for conformational sampling, had higher aggregation propensity (234). Subsequent studies on Aβ have identified oligomeric intermediates that undergo noteworthy conformational transitions; molecular dynamics simulations of Aβ40 performed by Xu et al. identified a mixed α-helix/β-sheet–rich species in the oligomerization process. Additionally, the authors also observed a gradual increase in β-sheet content as high molecular weight species emerged and postulate transition of the Aβ random coil to an α-helical intermediate, with the eventual appearance of β-sheet structure (92). The investigators also conclude that significant secondary structural changes accompany the oligomerization of Aβ, but remain unsure whether these transitions occur in the monomer prior to assembly or due to structural rearrangements in the nascent oligomer (92). A prior computational study on Aβ42 oligomerization also revealed that the N-terminal region of the protein retains a significant degree of disorder, even as the rest of the protein transitions to an amyloid form (235). The N-terminal residues from amino acids 1 to 6 were extended far from the hydrophobic core of the incipient amyloid structure, a feature that was common among Aβ40 and Aβ42 oligomers (235). This observation was further corroborated by Yang and Teplow, who showed that the N-terminal region of Aβ42 does not attain any order and remains as a random coil without contacting the central or C-terminal region of the protein during amyloid formation (236). Using mass spectrometry, Lieblein *et al*. identified two distinct oligomeric states of Aβ42 that differ in their conformation: a compact state and an extended state (237). Importantly, the authors observed that tetrameric oligomers of Aβ are unstable and unstructured to sustain propagation but form a stable nucleus upon transition to a pentamer and higher (237). Echoing these results, Barz et al. found that oligomeric conformers of Aβ40 and Aβ42 that exists in an extended state display higher aggregation propensity, while compact oligomeric conformations are more stable (238). The authors also remark that the N-terminal regions of both proteins are exposed to solvent and potentially engage in interaction with ligands (238). Overall, the tetrameric form of Aβ seems to represent the cusp from which the aggregation either proceeds on-pathway to fibrils or is routed to the off-pathway (238, 239, 240). The precise factors that govern the pathway chosen are unclear, but environmental factors seem to play a role (12, 239, 240). The dynamic nature of early Aβ oligomers is evident from these results, but ambiguity remains with respect to the details surrounding their transition to the ordered and rigid structure that eventually forms.

#### Fibril structure

The identification of atomic-resolution structures of Aβ fibrils has revealed the presence of polymorphic assemblies (153, 154, 155, 156, 241, 242). The structural elucidation of all these fibrils was performed *via* cryo-EM (153, 154, 155, 156, 241, 242). In the work by Kollmer *et al*., the AD brain–derived Aβ40 fibrils reveal three distinct morphological species dubbed as I, II, and III (241). All three fibrils display a right-handed twist but differ in their cross-over lengths and widths (241). Morphology I was characterized in detail and has a C-shaped overall fold, with N- and C-termini forming arches around the central core. In line with studies on oligomers, the N terminus of the fibril shows noteworthy solvent exposure (241). The right-handed twist of these fibrils is novel and differed from Aβ40 fibrils formed *in vitro* (241). In another study by Lu *et al*., Aβ40 fibrils isolated from two different patients showed polymorphism differentiated by an aperiodic twist (243). The fibrils showed striking differences in their overall geometry; fibrils from one patient adopted a two-fold symmetry, while the other polymorph had a three-fold symmetry (243). It is not surprising then that both patients had different diagnoses, Lewy body dementia, and AD, although both showed prominent plaque deposition (243). The 3D structure of Aβ42 fibril shows a gross similarity to its Aβ40 counterpart in that the protofilaments form intertwined assemblages (155), a common trend in many amyloid proteins. The fibril core is composed of residues 18 to 42, which form two parallel, β-strands (β1:18–26 and β2: 31–42) (155), while the rest of the protein (amino acid 1–17) is unstructured (155). The authors also identified a salt bridge between D23 and K28 (155) and hydrophobic interaction between residues F19 and G38 that connect the two β-strands of the core (155). In the structure of Aβ42 fibrils elucidated by Gremer *et al*., they identified the entire sequence of the protein engaged in amyloid formation, including the unstructured N terminus (244). The overall fold of the amyloid forms a shape resembling the letters ‘LS’e, as viewed parallel to fibril axis (244). The ends of the fibril present a regular, helical symmetry, which acts as an interface where monomer addition can take place (244). These morphologies are drastically different from those observed in other AD relevant fibril structures (153, 156). Additionally, Scherpelz et al. propagated Aβ fibrillar seeds from the brain parenchyma and vasculature, identifying polymorphism within the two species (245). In another study, Yang and co-workers identified two structural dsitinct filaments of Aβ42, which adopted an S-shaped fold (246). Of note is the finding that the filaments from sporadic and familial AD differed from each other (246).

### αSyn: oligomer dynamics

Experimental studies on αSyn aggregation by Cremades *et al*. and follow up work by Iljina et al. identified two conformationally distinct assemblies of αSyn oligomers using a FRET assay; these were dubbed as low-FRET and high-FRET oligomers (247, 248). The high-FRET oligomers displayed more resistance to proteolytic digestion, had higher stability to environmental fluxes, and exhibited greater cytotoxicity in comparison to the low-FRET oligomers (247, 248). This result suggests that the former represent a more mature, potentially on-pathway oligomeric assembly, while the latter is composed of early stage, nascent aggregates (247, 248). The authors also suggest potential conversion of low-FRET species into the high-FRET oligomers postconformational rearrangement (247, 248). The higher sensitivity of the low-FRET oligomers to environmental changes and proteolytic digestion also indirectly hints at the prevalence of structural disorder within these species. Support for these findings comes from computational modeling of αSyn aggregation which reveals that the energy landscape permits the presence of two distinct populations of αSyn: disordered oligomers with a weaker hydrogen bond network and prefibrillar oligomers which show an ordered assembly with numerous interchain, parallel hydrogen bonds (249). Furthermore, these simulations were unable to clearly demarcate the energy barrier between the two populations suggesting easier interconversion. Additionally, the investigators also identified a stable nucleus size of two to three monomeric units for αSyn oligomers, which agrees well with experimental observations (249, 250, 251, 252). Significant dissociation and reassociation events were also seen in the early oligomeric species, which potentially provide a route for transition and interconversion of distinct oligomeric forms (249). Using hydrogen-deuterium exchange coupled mass spectrometry, Mysling *et al*. were able to locate the disorder in αSyn oligomers to the C-terminus region based on its rapid isotopic exchange with the surrounding solvent (253). Surprisingly, the central amino acids 55 to 76 also showed slow isotopic exchange, suggesting they are solvent exposed to a certain degree (253). This suggests that further conformational conversions take place from the species investigated to the final fibrillar form, where these residues are protected (254). In another study by Zhou *et al*., the investigators utilized atomic force microscope-infrared spectroscopy to monitor morphological and secondary structural transitions in αSyn aggregates (255). The results show that αSyn aggregation proceeds in a manner not dissimilar to Aβ; there is a transition from a disordered random coil to an α-helical intermediate, which transitions to the β-sheet–rich structure (255). The authors also conclude that there are structural rearrangements from an antiparallel β-sheet to a parallel one (255). Delving deeper into the dynamics and structural transitions of αSyn aggregation, Chen *et al*. used simulation and *in vitro* assays to identify two distinct αSyn oligomer populations which differed in their secondary structural characteristics (256).

#### Fibril structure

The fibrillar structure of αSyn identified by Tuttle *et al*. using solid-state NMR spectroscopy reveals a new Greek-key topology adopted by the parallel, in-register β-sheet (169). This topology is replicated in the fibrils investigated by Li *et al.* using cryo-EM, who also saw an orthogonal Greek-key motif of αSyn fibrils (170). This Greek-key topology, which is formed by the strands of β-sheet, is named as such as because the overall protein fold resembles the pattern seen on ancient Greek pottery. The fibrils engage the same residues in generating the core, extending from aa 37-99 (169, 170). Both these fibrillar structures are generated from a dimeric interface formed by the central amino acids (from ∼50–60) within the non-amyloid-beta component region (169, 170). The sequence spanning the amyloid core also corresponds to mutations associated with familial PD, suggesting a potential source of conformational variety among the fibrillar forms. In another study by Li *et al*., where cryo-EM was used, we see an occurrence of the common structural motif of the Greek-key (171). The investigators identified two distinct polymorphic structures they termed as ‘rod polymorph’ and ‘twister polymorph’ (171). We also see molecular-level evidence of polymorphic αSyn strains based on secondary structure, morphology, and cytotoxicity assays (257, 258, 259). Structural elucidation of fibrils formed by αSyn mutants associated with familial PD show distinctive structural conformations and so do the fibrils generated under different conditions (170, 171). In another cryo-EM investigation of αSyn fibril structure by Guerrero-Ferreira *et al*., the Greek-key motif can be identified again and the protofilaments of the fibrils show a left handed helical twist (172). Additionally, Yang and co-workers report that αSyn filaments isolated from the brain of individuals afflicted with PD, Parkinon disease dementia (PDD), and DLB adopt a single fold, termed as the Lewy fold (260). Imporantly, the authors conclude that this fold deviates from that adopted by αSyn filaments in patients with MSA (258, 260).

### Tau 2N4R: oligomer dynamics

With the use of hydrogen-deuterium exchange coupled mass spectrometry, Huang *et al*. probed the conformational dynamics of soluble Tau aggregates (261). The results indicated that the third repeat of MTBR (amino acid 308–315) was protected from isotope exchange in the aggregated form, although in the monomeric form, it shows solvent exposure (261). This is consistent with the knowledge that this region forms the amyloid core and is expected to be solvent protected. Importantly, the authors identified that the extreme end of the C terminus and the N terminus of the oligomeric and fibrillar aggregates, along with monomers, showed similar levels of isotopic exchange, suggesting the persistence of intrinsic disorder within these regions (261). Using electron paramagnetic resonance (EPR) experiments, Eschmann *et al*. analyzed the aggregation dynamics of Tau in presence of heparin (262). Based on the findings, the authors suggest the existence of two conformational populations of Tau oligomers en route to fibrillation: a compact state and an extended state (262). The authors conclude that the extended conformation is more prone to aggregation, while the compact state resists it and that the interconversion between these species is the rate-limiting step for the process (262). The authors speculate that environmental factors and conditions may act as triggers that might induce such a transition (262). To probe conformational changes associated with Tau aggregation, Lasagna-Reeves *et al.* used an immunochemical approach (263). The investigators used a conformation-dependent antibody sensitive to toxic Tau oligomers and were able to detect them in early stages of AD pathology and not afterward (263). These results suggest that early pathological changes in AD might be induced by a toxic Tau conformer which converts to a fibrillar form with attenuated toxicity.

#### Fibril structure

Polymorphic aggregates of Tau isoforms are highly prominent in various Tauopathies (183, 185, 186, 264, 265). Like fibrils of other proteins, structural elucidation of Tau fibrils was performed using cryo-EM (185, 186, 264, 265). Using cryo-EM, Fitzpatrick *et al.* investigated the paired helical filaments and straight filaments of Tau that are prominent in AD (188). The investigators identified that the fibril core of both polymorphs is made of the same amino acids (from 306 to 378) and adopt the same ‘C’ shaped fold (186). Additionally, both protofilaments are composed of eight β-strands (β1 - β8). The results reveal that the morphological differences between the polymorphs are down to the different packing of their protofilaments (186). Both protofilaments engage different salt bridge interactions and hydrogen bond network leading to different contacts at their interface (186). Finally, the authors also identified ‘fuzzy coats’ (disordered regions) flanking the amyloid cores of both polymorphs (186). In structural studies by Falcon *et al*. on the Tau fibrils derived from chronic traumatic encephalopathy (CTE), a novel polymorphic fold is observed (185). The authors investigated fibrils from three different cases with documented CTE and found that they were similar in all three cases but differed from fibrils of Tau identified in AD (185). This result is significant because the known risk factor in this disorder is repeated head trauma, which is postulated to induce widespread chronic inflammation (266). Such association between physical injury, inflammatory insults, and Tau polymorphism highlights a potential underlying link. In a recent report, Shi *et al*. revealed a novel, three-layered fold adopted by Tau filaments from progressive supranuclear palsy (265). The authors also suggest a hierarchical classification of different Tau polymorphs based on the regions involved in forming the amyloid core. The R3, R4 pseudo-repeats, along with 10 to 13 residues from the C terminal form the amyloid core in all structures identified thus far, with differences lying in the N-terminal region (265). This classification highlights the striking differences of Tau polymorphs within different diseases. In AD and CTE, the R3, R4, and C-terminal residues are involved in amyloid core formation, and in corticobasal degeneration (CBD), the core spans the R2, as well (265). Within progressive supranuclear palsy, the same regions as the CBD filaments are involved, but there is noteworthy difference within the overall fold and symmetry of the structures (265). A recent study by Dregni et al. showed that there is conformational compatibility between the misfolded forms of Tau 3R and 4R (267). Additionally, the investigators also amplified AD brain-derived Tau filaments using a mixture of 3R and 4R isoform monomers and found that the pathological AD seed recruits both isoforms seamlessly in a 3:2 ratio of 4R:3R. This finding suggests that a similar energy landscape exists for the aggregation of both isoforms and that the structural rearrangements they undergo upon assembly are not drastically different. The work of Zhang *et al*. compared the fibril structures of 3R and 4R Tau generated *in vitro* in the presence of heparin to those derived from AD or FTD (183). The investigators identified that the *in vitro*–generated fibrils adopt distinct folds and engage different repeats in their core (183). These results are significant as they underline the influence of the environment on the structural variety of fibrils.

### TDP-43: oligomer dynamics

The dynamics of TDP-43 aggregation remain poorly investigated. *In vitro* experimental studies on the full-length protein are hampered by poor solubility in experimental conditions (268, 269). Still, indirect evidence parallels the results seen with other amyloid proteins thus far in that structural, morphological rearrangements are observed with TDP-43 aggregation as well (268, 270). In immunochemical and biophysical studies conducted by Fang *et al*., TDP-43 oligomers were found to lack a significant amount of β-sheet structure (270). TDP-43, although is closely implicated in multiple pathologies, is poorly characterized, and this is evident in the lack of information on its oligomers and other aggregation intermediates.

#### Fibril structure

The structure of TDP-43 filaments isolated from ALS-FTD–afflicted individual shows an amyloid-like core spanning 79 residues from amino acid 282 to 360, corresponding to the LCD of the protein, which is enriched in glycine residues (201). The investigators used cryo-EM for these studies and found that the parallel, in-register β-sheet of TDP-43 is composed of ten β-strands and adopts a novel overall fold which resembles a double spiral (201). The filaments also showed a right-handed helical twist (201). The authors also note that no phosphorylation sites are localized to the amyloid core, where all the serine residues (except S342) are buried (201). The results also reveal the presence of a unique, solvent-exposed polar surface on the C-terminal end of the amyloid core that the authors comment is not observed in any other amyloid fibril (201). Notably, these filaments lack grooves that can serve as binding sites for ligands, potentially explaining the poor thioflavin-T/S binding of these structures (201). The authors noted that these filaments bear no resemblance to TDP-43 fibrils generated *in vitro* (201). Importantly, the glycine-dense sequence of the amyloid core region gives to a high number of turns and inhibits the adoption of the hallmark cross-β sheet structure of most amyloid, instead being similar to that observed in FUS and heterogeneous nuclear ribonucleoproteins (hnRNPs) (201). The authors note that the fibrils isolated from different brain region and from different patients show no differences (201). With respect to TDP-43 strains, there is evidence from proteolysis assays that TDP-43 aggregates from different FTD subtypes (subtype A, B, and C) have unique sensitivities and prion-like seeding activities (201, 271).

## Conclusion

From the results discussed so far, it is clear that there is an enormous conformational variety in amyloid structures, from metastable oligomeric species to the rigid polymorphic fibrils. The relevance of this conformational variation to unique pathological sequalae has only recently been embraced within the field of amyloid studies. Crucially, it is now recognized that these conformers need to be characterized as a preliminary step towards the development of any therapeutic strategies. As reviewed here, the majority of amyloidogenic proteins are unstructured or contain regions that are unstructured. This fact has pathophysiological relevance, as it allows the proteins to serve multiple physiological roles, but it is also found to engender an enhanced aggregation propensity. The crowded intracellular milieu adds to the complexity of characterizing amyloid polymorphism if one considers transient interactions and environmental conditions as prominent modulators of protein conformation. Among the many factors that influence protein conformation, environmental pH is observed to have a significant role; Aβ, Tau, and αSyn all are shown to undergo structural changes in response to pH as the charge states of the acidic and basic residues are altered (15, 60, 127, 272, 273, 274, 275). The presence of metal ions or free radicals are also observed to induce potent changes in protein structure (276, 277, 278). Additionally, amyloid proteins are known to interact with a multitude of biomolecules, such as other proteins (193, 279, 280, 281, 282), nucleic acids (209, 282, 283, 284), lipids (285, 286), biochemical compounds, including osmolytes (287) and sugar moieties (288). Furthermore, the monomeric and aggregated form of proteins can have their intrinsic properties modified in the form of PTMs, mutations, and truncations. Another factor that might reveal itself to play a significant role in modulating amyloid formation and structure is the phenomenon of liquid-liquid phase separation or biomolecular condensation (289). Studies in the past decade have shown that this process is ubiquitously utilized by the cellular machinery in mediating various signaling pathways, biochemical reactions, metabolic steps, and more (289). In this process, cellular components such as biomolecules (including proteins and nucleic acids) transition from a mixed, soluble state to a distinct phase that is separate from their surroundings (289). An excellent analogy for visualizing this process is to imagine the presence of distinct droplets of oil in water. Exactly what role this process plays in inducing amyloid aggregation is currently the focus of intense investigations, most of which are *in vitro*. It will be interesting to see these results and to then be able to understand how this might affect structural polymorphism of amyloids.

**Figure 3:**
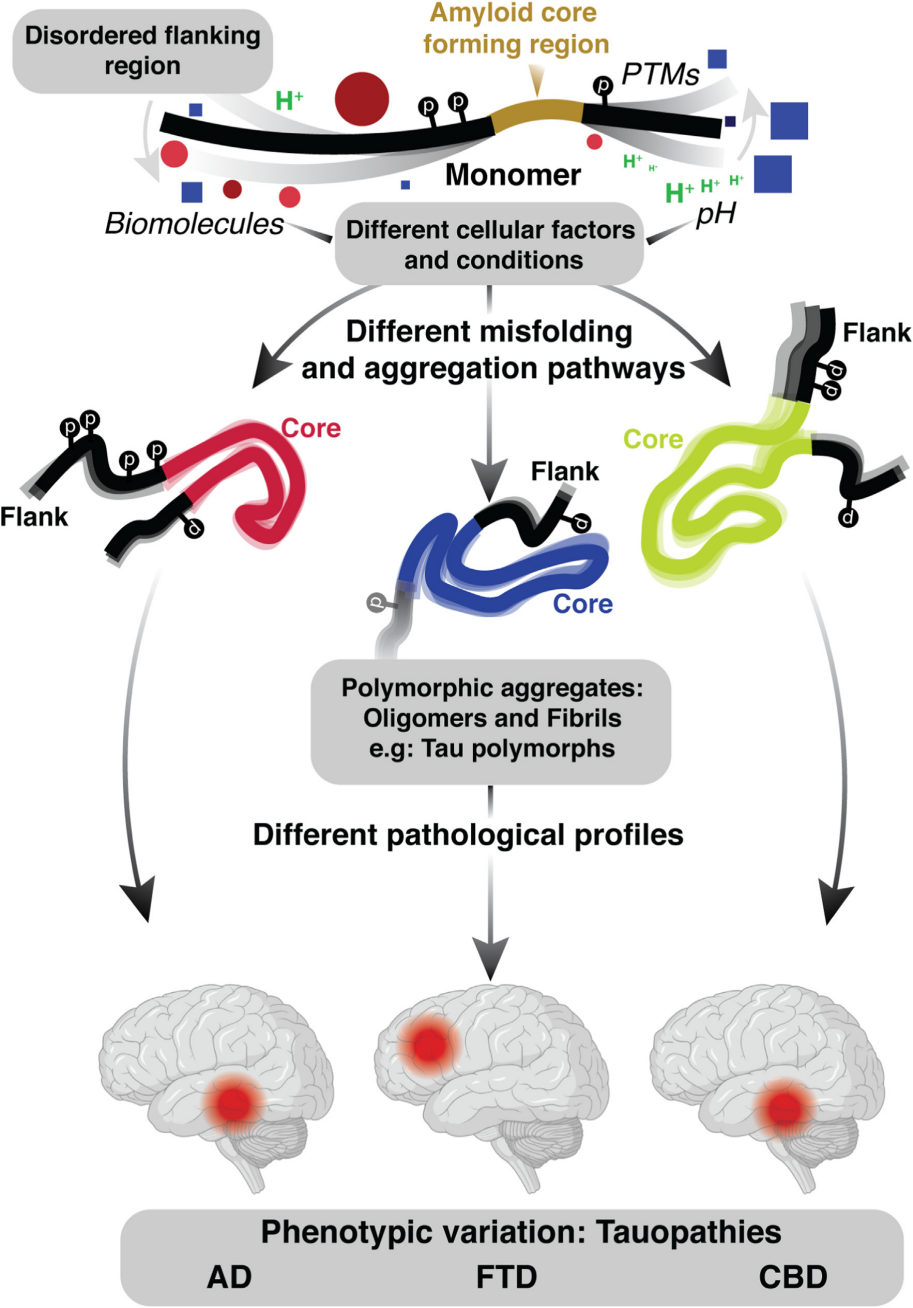
**Disorder and polymorphism in amyloid proteins**. Structurally disordered segments (*black*) flanking the eventual core of aggregates in amyloid proteins (*gold*) interact with various biomolecules and cellular factors such as pH (H+) (60), nucleic acids (316), other proteins (317), etc. (Indicated by *green*; protons, *yellow star*; free radicals, biomolecules; *red circles*, *blue boxes*). Such interactions, along with PTMs (139) such as phosphorylation (p), prominent in case of Tau, can modulate the conformational ensemble of the monomer and induce polymorphism in aggregates which underlies phenotypic variations observed in pathology. In Tauopathies, the aggregates of Tau are polymorphic in Alzheimer disease (186), frontotemporal dementia (184), and corticobasal degeneration (264). Figure is generated with the help of BioRender. PTM, posttranslational modification.

As depicted in the schematic (Fig. 3), we propose that events which occur within the early misfolding of the monomer and incipient oligomers generate distinct polymorphic aggregates. As stated by Fandrich *et al*. (42), if we extend the parallels between molecular crystallization and protein aggregation beyond kinetics, then polymorphism of proteins can be similarly viewed as a consequence of initial conditions. The events and interactions in these initial conditions can modulate the aggregation pathways accessible to the innate assemblies, routing them to an aggregated state with a distinct structural identity (Fig. 1). In neurodegenerative diseases, each polymorph has a unique pathological profile which can activate distinct toxic sequalae, giving rise to phenotypic variations (Fig. 3). For example, the polymorphic aggregates of Tau that are found in AD, FTD, and CBD differ significantly from one another (Fig. 3).

The fact that early aggregation intermediates have such consequential roles in dictating pathology identifies them as potential targets for therapeutic strategies. Current therapeutic tools utilize three strategies to inhibit or attenuate protein aggregation (290, 291, 292, 293, 294): (i) inhibiting aggregation, (ii) catalyzing the disassembly of aggregates, and (iii) routing protein aggregates to nontoxic conformation. Additionally, strategies have aimed to enhance clearance of aggregates or decrease the generation of amyloid proteins (295). The flanking regions of amyloid cores can also be considered as promising therapeutic targets because of the following reasons: (i) their almost ubiquitous presence in amyloid proteins, (ii) the presence of interaction modules (MoRFs, SLiMs, LCDs), allowing them to interact with a wide array of molecules (296), and (iii) their important ability to maintain high solvent exposure, even in aggregated forms of proteins, enabling easier accessibility for any drug molecule (110, 296). Additionally, our knowledge regarding the interaction partners of disordered interaction modules is constantly growing (296), and with the advent of neural networks and artificial intelligence, our predictive capabilities for structural properties of disordered regions are also becoming more reliable (146, 147). These strategies can allow us to modulate the structural ensemble of disordered proteins and potentially redirect the aggregates to a state with attenuated toxicity. Currently, the most promising disease-modifying tools we have are immunotherapeutic approaches that target amyloid aggregates with antibodies (297, 298), highlighted by the recent Food and Drug Administration (FDA) approval for anti-Aβ immunotherapy (299, 300). There is excitement and caution among scientists and clinicians in the field with regards to some of these recent developments (301); aducanumab is a monoclonal antibody targeted against the aggregated form of Aβ (oligomers and fibrils), which aims at their clearance by binding to them and eliciting an immune response. The clinical trial results so far have shown only marginal benefits in improving cognitive decline in patents with prodromal or mild-AD, generating widespread concern upon its approval by the FDA (301). Recently, another monoclonal antibody directed against Aβ aggregates (oligomers, protofibrils, and fibrils), lecanemab, was approved by FDA as a disease modifying agent for AD (299). Considering our growing understanding of polymorphic amyloid structures, it seems likely that antibodies targeting generic aggregates of amyloid proteins will continue to perform poorly in therapeutic applications. Accordingly, focus has shifted to investigating conformation-specific antibodies that target polymorphic structures (302, 303, 304, 305, 306, 307). Exciting results from *in vitro* experiments discussed thus far suggest that the conformational ensemble of monomers can be modulated by various additives and environmental condition (308, 309). One can then imagine a battery of additives being developed that can reroute the aggregation pathway to an inert final form (294, 310, 311). Additionally, antibodies binding to the flanking regions might abrogate aggregation altogether, that is, antibodies engineered to target and stabilize certain nontoxic structures in amyloid-flanking regions can provide a fruitful avenue. Such a strategy is already being tested with polyphenolic compounds, such as curcumin (found in turmeric) and its derivatives, which have shown potential in *in vitro* studies (312, 313, 314).

Overall, we aimed to highlight the not-so passive role of flanking regions of amyloid cores in pathology. These flanking regions seem to play a role in the conformational fluctuations of the amyloid protein, modulating its end structure. In this context, some outstanding questions that arise are: (i) to what extent does the solvent exposure and conformational dynamics of flanking regions differ between the oligomeric and fibrillar forms, (ii) if such changes do occur, are they the result or the cause of the final structure observed. In a similar vein, it remains to be definitively established whether PTMs and other biochemical interactions that the flanking region engages in are the cause of oligomeric and fibrillar conformations or are consequential. Based on available evidence, we suspect that the former is true in most cases. Furthermore, we are also lacking a detailed analysis of whether the amount of disorder in flanking regions of an amyloid protein correlates with the extent of fibrillar polymorphism. Due to their many interactions and possible modifications, the amyloid flanking regions can, in effect, dictate disease progression and severity. It is thus imperative we probe these regions further to pinpoint the source of polymorphic behavior. We hope that the recent understating with respect to structural disorder in amyloid proteins that we have outlined here can help guide any future investigations and be translated into the identification and development of effective drugs.

## Conflict of interest

The authors declare that they have no conflicts of interest with the contents of this article.
